# Microbial Biofertilizers for Salinity Stress Mitigation in Hydroponic Systems

**DOI:** 10.3390/cimb47121029

**Published:** 2025-12-10

**Authors:** Prabhaharan Renganathan, Lira A. Gaysina, Edgar Omar Rueda-Puente

**Affiliations:** 1 Department of Bioecology and Biological Education, M. Akmullah Bashkir State Pedagogical University, 450000 Ufa, Russia; prabhaharan06@gmail.com (P.R.); lira.gaisina@gmail.com (L.A.G.); 2 All-Russian Research Institute of Phytopathology, 143050 Bolshye Vyazemy, Russia; 3 Phystech School of Biological and Medical Physics, Moscow Institute of Physics and Technology, 141701 Dolgoprudny, Russia; 4 Departamento de Agricultura y Ganadería, Universidad de Sonora, Blvd. Luis Encinas y Rosales, Hermosillo 83000, Sonora, Mexico

**Keywords:** plant growth-promoting bacteria, ionic homeostasis, exopolysaccharides, ACC deaminase, encapsulation technology, hydroponics

## Abstract

Salinity accumulation is a critical abiotic constraint in hydroponic agriculture, particularly in recirculating systems, where limited leaching and nutrient cycling intensify ionic accumulation and increase the conductivity of nutrient solutions. Hydroponic crops are sensitive to osmotic and ionic stress, which leads to reduced water uptake, disrupted nutrient homeostasis, and yield loss. Traditional mitigation strategies, such as nutrient dilution, flushing, and water blending, provide temporary relief while increasing operational costs, nutrient discharge, and water consumption. Microbial biofertilizers, including plant growth-promoting bacteria, fungi, and microalgae, offer a sustainable approach for enhancing salinity resilience. These microorganisms influence root zone processes through mechanisms such as ion transport regulation, exopolysaccharide-mediated Na^+^ immobilization, osmolyte accumulation, antioxidant enhancement, phytohormonal modulation, and siderophore-mediated micronutrient mobilization. This review (i) summarizes the physiological, microbial, and system-level drivers of salinity stress in hydroponics, (ii) synthesizes evidence for microbial inoculation in saline solutions, and (iii) identifies research gaps related to formulation stability, disinfection compatibility, and commercial-scale validation. We address advances in hydroponic microbiology, emphasizing optimized delivery systems, including encapsulated formulations, consortium-based inoculation, and system-specific strategies to support microbial colonization in soilless environments.

## 1. Introduction

Hydroponics have become an important component of modern controlled-environment agriculture owing to their high resource-use efficiency, environmental sustainability, and precise nutrient management. Unlike soil-based cultivation, hydroponics uses an inert medium or direct nutrient solution that enables optimal nutrient availability and water utilization [1]. The transition towards soilless cultivation systems is driven by land degradation, water scarcity, urban population growth, and demand for pesticide-free production. Hydroponic systems offer several advantages, including enhanced nutrient use efficiency (NUE) and resource conservation [2,3,4]. However, they remain susceptible to physicochemical imbalances, particularly salinity accumulation, owing to the absence of soil-mediated buffering mechanisms [5,6].

Salinity stress is a major abiotic limitation in hydroponic crop production because increased electrical conductivity (EC) affects physiological and biochemical processes [7,8]. Salinity has two phases: an osmotic phase that inhibits water uptake and growth, and an ionic phase with excessive Na^+^ and Cl^−^ accumulation, which interferes with the uptake of essential nutrients, including K^+^, Ca^2+^, and Mg^2+^ [9,10]. These disruptions affect stomatal conductance, photosynthetic capacity, chloroplast structure, enzymatic activity, and metabolic pathways, reducing biomass and yield potential [11]. Hydroponic vegetables, such as lettuce, spinach, cucumber, and strawberries, have defined EC tolerance thresholds and exhibit substantial yield reductions under moderate salinity [12,13,14,15,16,17]. For instance, a unit increase in EC beyond the optimal threshold can decrease productivity by 10–15% [18], making salinity management critical in intensive production systems [19,20,21].

Hydroponic systems, particularly the nutrient film technique (NFT), deep-water culture (DWC), and recirculatory systems, experience changes in nutrient composition over time [2]. Moreover, ionic gradient formation and heterogeneous salt distribution occur in substrate-based hydroponics, with lateral EC variations in coconut coir lettuce systems [22]. Real-time monitoring and automated sensing technologies are crucial for maintaining salinity stability in recirculating systems [23,24]. Conventional salinity mitigation includes solution dilution, partial nutrient replacement, flushing, and improved water quality management [25]. Although these approaches provide temporary relief, they lead to nutrient wastage, increased operational costs, and reduced sustainability under water-limited conditions [26]. Consequently, the focus has shifted towards biological interventions that enhance plant resilience under saline conditions.

Plant growth-promoting microorganisms (PGPM) have emerged as biotechnological tools for enhancing salinity tolerance owing to their physiological roles in plants. Studies have shown that plant growth-promoting bacteria (PGPB) regulate membrane transporters, including SOS1, HKT1, and NHX antiporters, reducing Na^+^ toxicity and maintaining K^+^/Na^+^ homeostasis [27,28,29,30]. Biofilm-forming microbes stabilize the rhizosphere [31,32], and microbial modulation supports cellular homeostasis under saline conditions [8,33]. ACC-deaminase-producing microorganisms reduce ethylene levels and enhance root elongation [34,35,36]. Microbial phytohormones, siderophores, and volatile organic compounds (VOCs) facilitate plant stability in hydroponics [37,38,39].

Despite substantial evidence supporting microbial inoculation, hydroponic environments limit microbial colonization, survival, and functional efficacy. Sanitization treatments, such as UV-C, ozone, and hydrogen peroxide, significantly reduce microbial viability [40,41,42]. Additionally, hydraulic turbulence and low organic carbon availability require optimized delivery strategies, including encapsulation and carrier matrices [43,44]. Encapsulated inoculants show improved durability, enhanced colonization, and salinity mitigation potential. However, commercial-scale validations remain limited, standardized inoculum application protocols are underdeveloped, compatibility with routine disinfection practices is poorly understood, and the integration of biological inputs into automated fertigation systems requires further advancement. This review synthesizes the physiological, microbial, formulation, and system-level perspectives of salinity management in hydroponics, addressing research gaps in sustainable biological salinity mitigation.

## 2. Salinity Stress in Hydroponics

### 2.1. Physiological Responses and Mechanisms of Salinity Stress in Hydroponic Crops

Salinity is a major abiotic stress factor in hydroponic crop production, as increased ionic strength affects nutrient balance, plant–water relations, and cellular metabolism. Although hydroponic systems have NUE, their closed recirculatory nature makes them prone to ion accumulation owing to limited leaching and the absence of soil-based buffering [2,5]. Ionic inputs from water, fertilizers, and evapotranspiration increase EC, causing osmotic and ionic disruptions in the growth medium [6]. Studies have shown that an increase in EC (2–3 dS·m^−1^) reduces leaf expansion and shoot growth in recirculating hydroponics [7]. Root exposure to ions intensifies osmotic constraints compared to soil systems, which provide adsorption, diffusion, and buffering functions [8].

Salinity stress induces physiological disturbances in two functional stages. The osmotic stage occurs when increased EC limits hydraulic flow and reduces turgor, stomatal conductance, leaf expansion, and photosynthetic rate. The ionic stage develops when Na^+^ and Cl^−^ accumulate and interfere with K^+^, Ca^2+^, and Mg^2+^ uptake and transport, altering nutrient homeostasis and metabolic pathways. These antagonisms inhibit photosystem stability and reduce carbon assimilation efficiency [9,11]. Hydroponic barley exposed to 100–200 mM NaCl showed high Na^+^ retention and reduced K^+^/Na^+^ ratios, highlighting the importance of regulating ion exclusion, vacuolar sequestration, and membrane transport in soilless systems [10].

Salinity-induced oxidative stress is a secondary limitation because continuous ion exposure enhances ROS generation and metabolic instability. Excessive ROSs damage lipids, pigments, proteins, and nucleic acids, compromising cellular integrity [2]. Studies have shown that 50 mM NaCl can inhibit antioxidant enzymes, including SOD, CAT, and APX, increasing oxidative stress [8]. Furthermore, rapid biochemical changes within 24 h, with proline accumulation, indicate metabolic reorientation towards osmolyte protection and ROS mitigation to maintain cellular function [45]. These responses highlight the importance of maintaining ionic equilibrium, membrane stability, and antioxidant defenses in hydroponic saline environments.

### 2.2. Crop-Specific Salinity Sensitivity, Thresholds and Productivity Losses

Salinity tolerance in hydroponic crops varies among species, with a narrow optimal EC range for most leafy and fruiting vegetables grown hydroponically. In lettuce, optimal growth occurs at 1.2–1.8 dS m^−1^, whereas EC levels above 2.0 dS m^−1^ reduce biomass and chlorophyll content, indicating stress-mediated impairment of photosynthetic and nutritional performance [12]. Studies have shown a 50% reduction in fresh biomass at 3.0 dS m^−1^ NaCl-induced EC [13]. Similarly, Akter et al. [14] observed a 75–77% yield reduction in butterhead lettuce when EC increased from 2.5 to 6.0 dS m^−1^ compared with the optimal 1.5–2.0 dS m^−1^.

In fruiting vegetables such as tomatoes, salinity tolerance is moderate, with a 25–30% yield reduction at EC above 6.0 dS m^−1^, showing leaf ion toxicity and decreased photosynthetic activity [15]. EC increases from 2.5 to 9.5 dS m^−1^ reduced tomato yield by 31%, although fruit firmness and soluble solids may increase owing to osmotic concentration [11]. Similarly, cucumbers exhibit optimal performance at 2.0–3.5 dS m^−1^, whereas strawberries (*Fragaria* × *ananassa*) show a 20% yield loss at an EC of 2.0 dS m^−1^, indicating that certain horticultural crops possess lower thresholds for ionic accumulation under hydroponic conditions [16,17].

Meta-analyses show that for hydroponic vegetables, each 1 dS m^−1^ increase above optimum reduces productivity by 10–15%, indicating the importance of maintaining consistent EC levels for commercial viability [18]. This is related to limited ion uptake, translocation efficiency, and physiological stress responses. The hydroponic EC thresholds, yield reductions, and associated quality responses are presented in Table 1.

Economically, salinity-induced yield losses significantly affect high-technology greenhouse systems. For instance, tomato systems targeting 40 kg m^−2^ yields against a European production reference of 60–70 kg m^−2^ at €1.50 kg^−1^ experience an estimated €6 m^2^ revenue loss per cycle for every 10% yield reduction [19,20,21]. This emphasizes the importance of maintaining crop-specific EC thresholds to stabilize nutrient uptake and yield.

### 2.3. System-Level Salt Accumulation and Root-Zone Ionic Dynamics

Salt accumulation in hydroponic systems is controlled by system architecture and operational practices, which influence nutrient homeostasis and crop performance. In closed-loop systems, such as NFT and DWC, continuous recirculation promotes ionic accumulation owing to limited discharges [2]. Strawberry cultivation in closed systems without nutrient correction showed a 36% higher root-zone EC after eight weeks compared to systems with intermittent nutrient renewal [52]. Partial solution replacement leads to increased Na^+^ and Cl^−^ concentrations, whereas evaporative water loss concentrates the solutes. In hydroponic tomatoes, Na^+^ levels increased by 22 mmol L^−1^ over a 12-week period, showing 45% higher accumulation than in open-loop settings [23].

System-level ion accumulation depends on hydraulic closure, nutrient renewal frequency, and evapotranspiration. Closed-loop systems retain solutes for longer durations, and without periodic dilution, salts from irrigation water, stock solution impurities, and selective uptake increase EC. These patterns across crops demonstrate the influence of system design on salinity accumulation [11,53].

Substrate-based hydroponics, including perlite, rockwool, and coconut coir, exhibit spatial salt gradients. For instance, lettuce grown on coconut coir showed EC gradients, with lateral zones reaching 1.80 dS m^−1^ compared to 0.84 dS m^−1^ near the stems, indicating localized salinity from substrate drying and capillary redistribution [22]. Dense root mats in NFT channels can inhibit flow and reduce oxygen diffusion. Studies on closed tomato slabs have shown that drainage-based EC values do not represent the actual pore-water salinity. XGBoost models showed superior ECw estimation (R^2^ = 0.876; RMSE = 0.623 dS m^−1^) compared to Hilhorst conversion methods (R^2^ = 0.132; RMSE = 2.43 dS m^−1^) [24].

The ionic composition of nutrient solutions regulates the severity of stress. NaCl is the primary solute, whereas SO_4_^2−^ and HCO_3_^−^ interact with nutrient uptake and affect plant response. Na^+^ competes with K^+^ for transport, and Cl^−^ interferes with nitrate and phosphate uptake. These interactions affect pH, ionic strength, and solubility, reducing the availability of micronutrients [54]. Bicarbonate-rich solutions (>3 mmol L^−1^ HCO_3_^−^) reduce Fe availability and induce functional deficiencies, despite adequate Fe supplementation [55]. Therefore, salinity accumulation depends on system closure, substrate behavior, ion speciation, and microenvironmental heterogeneity, and requires targeted root-zone monitoring over bulk EC measurements.

### 2.4. Economic Impacts, Operational Risks and Biological Mitigation

Yield losses due to salinity cause economic losses in commercial hydroponics by affecting the crop quality and output. In hydroponic tomatoes, EC increased from 2.5 to 6.5 dS m^−1^ and reduced fruit yield by 29.9% [53]. Lettuce and cucumber showed significant yield reductions when EC exceeded 4 dS m^−1^, indicating narrow tolerance thresholds for these crops. High EC levels increase maintenance requirements through scaling and precipitation, requiring more frequent filter replacement, pipeline cleaning, and emitter servicing. Studies on greenhouse tomato systems have shown that net returns increase even with desalination-related water and energy costs [26].

The economic impact of salinity stress extends beyond yield reduction, as an increased EC intensifies the maintenance frequency, increases hydraulic component depreciation, and increases labor and input costs for nutrient solution management. These limitations indicate that salinity is both a physiological constraint and operational risk in commercial hydroponics.

Mitigation practices, including flushing or dilution, temporarily reduce EC but increase water use and nutrient discharge, creating a trade-off between water conservation and salinity control [25]. Closed-loop hydroponics can achieve 90% water savings and 60% fertilizer reduction compared with open systems [56]. These systems improve nutrient-use efficiency, although the benefits vary across crops and designs, and the efficiency depends on salinity control and system optimization [3,4]. However, its implementation is challenged by frequent EC fluctuations and difficulty in predicting ion accumulation under recirculation conditions.

Biological treatments reduce salinity-associated productivity decreases by strengthening plant physiological resilience rather than depending on salt removal. For example, mycorrhizal inoculation in DWC lettuce exposed to 80 mM NaCl increased shoot fresh weight by 72.5% compared to uninoculated plants [18]. Although significant studies exist on the physiological responses to salinity, research gaps remain in understanding system-specific ion accumulation, nutrient solution dynamics, and the integration of real-time EC and ion-sensing technologies. Current mitigation strategies are primarily chemical or physical and include flushing, dilution, and blending. Microbial biofertilizers enhance ion homeostasis, osmotic regulation, and antioxidant activity, reducing flushing frequency and stabilizing system performance [25]. Recent studies on halotolerant plant growth-promoting rhizobacteria (PGPR) and microbial consortia have shown that these inoculants can improve K^+^/Na^+^ selectivity, osmolyte biosynthesis, antioxidant enzyme activity, and salinity tolerance in hydroponically grown crops [57]. The economic sustainability of hydroponics depends on the integration of precise salinity management, nutrient recirculation, and microbial inoculation. Integrating hydroponic nutrient management with biological treatments provides more efficient mitigation than physical or chemical practices alone.

Physicochemical constraints explain why hydroponic systems respond poorly to ionic disequilibrium and why plants require biological buffering for physiological homeostasis. These limitations have motivated the adoption of microbial biofertilizers, whose attributes, such as ion transport regulation, osmotic adjustment, and redox modulation, operate at interfaces where salinity exerts its strongest effects. The next section examines how microbial processes antagonize ionic and metabolic disruptions, thereby offering a biological pathway for salinity mitigation in recirculating hydroponic systems.

## 3. Microbial Mechanisms of Salinity Mitigation

### 3.1. Ionic Homeostasis Mechanisms

Microbial biofertilizers enhance salinity tolerance through ionic regulation and maintenance of homeostasis. Under saline conditions, excess Na^+^ limits K^+^ uptake and affects the K^+^/Na^+^ ratio, enzymatic activity, and membrane stability. Halotolerant PGPR regulate ion transporter expression, maintain ionic equilibrium, and reduce salt stress in plants. Microbial inoculation activates SOS1 Na^+^/H^+^ antiporters, HKT1 high-affinity K^+^ transporters, and NHX vacuolar antiporters, reducing Na^+^ accumulation and improving K^+^/Na^+^ ratios in plants [27]. PGPR enhanced SOS1, HKT1, and NHX1 expression, limiting Na^+^ translocation to the shoots and improving K^+^ retention [28]. The SOS regulatory pathway coordinates these fluxes via the Ca^2+^ sensor SOS3 and stabilizes SOS1/HKT1 activity under saline conditions.

Hydroponic lettuce with *Bacillus subtilis* showed higher K^+^ uptake and improved K^+^/Na^+^ ratios under salinity, and DWC systems under 80 mM NaCl showed improved biomass and photosynthetic activity [18,30]. Halotolerant PGPR synthesize exopolysaccharides (EPS) that bind Na^+^ and reduce its activity in the rhizoplane, and many strains also carry Na^+^/H^+^ antiporter genes along with EPS biosynthetic operons [58]. EPS-producing microbes sequester Na^+^, decreasing its bioavailability near the root surface in hydroponic systems [28,59]. Biofilms formed on root surfaces immobilize Na^+^ within EPS structures while supporting water retention at the root–solution interface. Most halotolerant PGPB possess *nha* genes with EPS biosynthesis loci, and *B. subtilis* ES produced 4.7 g L^−1^ EPS at a 2% NaCl concentration [31,60]. *Pseudomonas aeruginosa* AG01 produced 0.89 g L^−1^ EPS under optimized conditions, and EPS-enriched biofilms enhanced microbial durability and ion sequestration in hydroponic systems [32,61]. EPS-mediated sequestration stabilizes microsalinity and hydration and creates protective microenvironments that improve ion transport and reduce ionic stress.

### 3.2. Osmotic Regulation Mechanisms

PGPR and mycorrhizal inoculants increased osmolyte accumulation, including proline, glycine betaine, and trehalose, maintaining cellular turgor, stabilizing proteins and membranes, and supporting water uptake under saline conditions. Hydroponic lettuce grown in 150 mM NaCl showed a 9-fold increase in proline accumulation, although a plant-derived biostimulant moderated this response [62]. Lettuce grown in 50 mM NaCl showed 22% higher proline levels and 41–42% lower malondialdehyde levels with PGPR inoculation [8]. Arbuscular mycorrhizal fungi (AMF) inoculation increases glycine betaine levels under saline conditions, and AMF-associated plants show 2-fold higher glycine betaine at increased NaCl levels [63,64]. These results show that ion transport regulation, EPS-mediated microsalinity buffering, and osmolyte accumulation operate synergistically to maintain ionic and osmotic balance in hydroponically grown crops under saline conditions.

### 3.3. Antioxidant and Redox Stabilization Mechanisms

Salinity-induced stress promotes oxidative stress through ROS accumulation, such as superoxide (O_2_•^−^), hydrogen peroxide (H_2_O_2_), and hydroxyl radicals, which damage membrane lipids, proteins, and nucleic acids, reducing membrane stability and PSII activity. Microbial biofertilizers enhance enzymatic (SOD, CAT, APX, GR, and POD) and non-enzymatic (ascorbate, glutathione, phenolics, and carotenoids) antioxidant systems and reduce oxidative markers such as malondialdehyde (MDA).

Hydroponic lettuce treated with PGPR-based biostimulants in 50 mM NaCl showed 2- to 7-fold increases in SOD, CAT, GR, and APX activities, whereas MDA levels decreased by 40%. In addition, they enhanced proline, phenolic compound, and chlorophyll content, stomatal conductance, and relative water content, indicating that microbe-mediated redox regulation aids osmotic resilience and photosynthetic stability [8]. Inoculation of lettuce and pak choi with beneficial microbes at 40–120 mM NaCl maintained biomass, leaf area, and pigment levels [18]. In DWC, *Bacillus*-based biostimulants (TNC Bactorr S13) at 20 mM NaCl enhanced growth, chlorophyll content, and gas exchange, while decreasing electrolyte leakage [39]. Similar physiological developments and antioxidant activation have been observed in various hydroponic crops, indicating a generalized mechanism of microbial redox assistance [33].

Microbial signals, including VOCs, siderophores, EPS, and phytohormones, regulate redox metabolism by modulating ethylene and abscisic acid (ABA) pathways, NADPH oxidase activity, and ROS-responsive transcription factors (TFs). Studies have shown that ACC deaminase- and phytohormone-producing PGPR reduce ethylene-associated stress responses, enhance antioxidant enzyme activity, and promote root development in high-EC systems [65]. Substrate-based systems have also shown that microbial inoculation stabilizes membrane function, limits oxidative damage, and enhances photosynthetic capacity under conditions of increased ionic strengths.

Siderophore secretion improves Fe availability under saline conditions, whereas Fe^3+^ precipitation inhibits the acquisition of micronutrients. Siderophore-producing rhizobacteria increase Fe solubility and its delivery to the roots, facilitating chlorophyll biosynthesis. A meta-analysis of 342 studies showed a 30% growth improvement, highlighting the central role of microbial Fe chelation under limited nutrient conditions [66]. In hydroponic systems, siderophore-mediated Fe mobilization correlates with increased chlorophyll content and biomass, particularly in leafy vegetables [65].

Floating hydroponic lettuce inoculated with microbial consortia (*Pseudomonas vancouverensis*, *Pseudomonas koreensis*, *Pseudomonas putida*, and *Pantoea agglomerans*) showed 26% higher leaf Fe, 2–4-fold higher chlorophyll a, b, and carotenoid levels, and 43% higher fresh biomass than uninoculated plants [37]. The reduced phenolic and anthocyanin content indicates that improved Fe acquisition reduces dependence on carbon-intensive antioxidant pathways. Under saline hydroponics, *Bacillus*-based PGPR improve K^+^/Na^+^ homeostasis, Fe nutrition, and chlorophyll retention while reducing chlorosis [39]. In NFT and DWC systems, siderophore-producing *Pseudomonas psychrotolerans* IALR632 increased shoot biomass by 15–55% and lateral roots by approximately 160%, supporting nutrient uptake and redox stability through improved root architecture [38].

### 3.4. Hormonal Signaling Mechanisms

Beneficial microorganisms regulate hormonal balance in plants under salinity, improving growth and physiological stability. Salinity enhances ethylene biosynthesis and senescence, and inhibits root elongation. PGPR-synthesizing ACC deaminase converts ACC into α-ketobutyrate and ammonia through acdS activity, reducing stress-induced ethylene accumulation [34,36]. *Bacillus* spp. with ACC deaminase activity can increase plant biomass under NaCl stress [35]. Soybeans exposed to 100 mM NaCl showed higher biomass, osmolyte content, antioxidant activity, and K^+^ retention when inoculated with ACC deaminase- and auxin-producing strains, *Pseudomonas pseudoalcaligenes* SRM-16 and *B. subtilis* SRM-3 [67]. Similarly, ACC deaminase-producing *P. putida* RT12 showed improved chlorophyll levels, relative water content, K^+^/Na^+^ ratio, soluble sugars, and antioxidants in 100–150 mM NaCl concentration [68]. In addition, microbial inoculants synthesize phytohormones, including auxins (IAA), gibberellins, and cytokinins, which promote root branching, shoot length, and nutrient assimilation in saline conditions. For instance, *Pseudomonas* strains modulate auxin distribution to enhance lateral root formation under saline conditions [69]. A commercial PGPR formulation (Nitrostim) increased fruit yield and quality in soilless tomatoes at 30 mM NaCl, and cherry tomatoes showed 53.2% higher yields [70,71].

Microbial VOCs modulate plant responses to ion stress through the production of compounds such as 2,3-butanediol, acetoin, and dimethyl disulfide. Under 100 mM NaCl, *Rahnella aquatilis* VOCs increased biomass by 1.3-fold, chlorophyll by 45.4%, and MDA by 58.5% [72]. Similarly, VOCs from *P. putida* enhanced peppermint biomass and root dry weight at 75–100 mM NaCl concentration [73]. Thus, VOC-mediated signaling, siderophore secretion, phytohormone production, and ACC deaminase activity form an integrated network that coordinates root–shoot signaling and systemic tolerance to high-salinity conditions. The association of microbial strains with their phytohormones, secondary metabolites, salinity conditions, host crops, and phenotypic responses is shown in Table 2. This dataset connects mechanistic understanding with applied utilization, facilitating the rationale for using hormonal and signaling formulations in the management of hydroponic salinity.

Several microbial mechanisms, including ion transporter regulation, EPS-mediated Na^+^ buffering, ACC deaminase activity, siderophore production, and VOC release, have been reported under saline conditions. However, their consistency varies among strains, crop species, and hydroponic settings. Studies on transporter modulation are often based on short-term expression rather than sustained ion homeostasis, whereas EPS benefits diminish in high-flow systems with weak rhizoplane retention. ACC-deaminase effects remain relatively efficient but are crop-dependent, and siderophore advantages decline when nutrient solutions already contain chelated Fe. VOC-mediated responses are primarily supported by controlled laboratory assays, with limited demonstration in recirculating hydroponics. These findings indicate that while microbial functions such as ionic regulation, EPS-mediated microsalinity buffering, antioxidant activation, and hormone modulation offer a mechanistic foundation for integrating microbial biofertilizers into hydroponic systems, their agronomic expression ultimately depends on system design, hydraulic conditions, inoculation strategy, and formulation stability.

**Figure 1:**
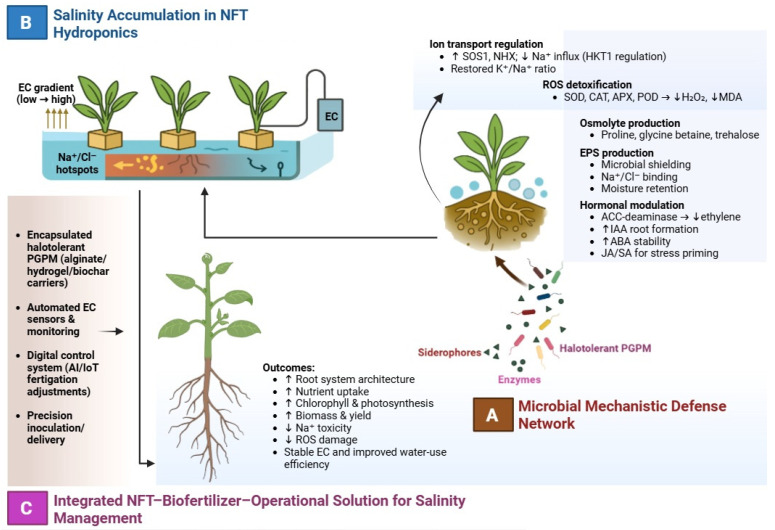
Integrated microbial and operational strategies for salinity mitigation in NFT hydroponics. (**A**) Key PGPM-mediated mechanisms, including ion transport regulation, oxidative stress reduction, osmolyte production, EPS-mediated Na^+^ buffering, and hormonal modulation. (**B**) Salinity accumulation in NFT systems showing EC gradients and Na^+^/Cl^−^ hotspots driven by nutrient solution recirculation. (**C**) Operational integration through encapsulated inoculants, automated EC monitoring, and artificial intelligence/internet of things-supported fertigation, improving nutrient uptake, root development, biomass, and yield under saline conditions. (↑) indicates a significant increase of the respective parameter, while (↓) indicates a reduction.

A summary of the integrated microbial, physiological, and system-level interactions indicating salinity mitigation in NFT hydroponics is presented in Figure 1.

## 4. Microbial Biofertilizer in Hydroponic Systems

### 4.1. Functional Performance and Plant Response Outcomes

Microbial biofertilizers, particularly PGPB, beneficial fungi, microalgae, and cyanobacteria, are emerging as a strategic approach for hydroponic salinity mitigation. Their functional performance has been demonstrated on inert substrates and in recirculating solution systems. Their effectiveness depends on the strain-level characteristics and operational compatibility with hydroponic conditions, including residence time, adhesion traits, and nutrient solution chemistry. Spore-forming Gram-positive bacteria, such as *Bacillus* spp., have shown superior performance owing to their durability, stress tolerance, and rapid colonization in hydroponic environments [18,33,39]. Bacterial–fungal consortia provide higher functional resilience under fluctuating EC conditions than single-strain inoculants [18]. Table 3 presents the quantitative responses, including yield gains, biomass accumulation, ion homeostasis, and antioxidants, in hydroponic crops inoculated with microbial strains under saline nutrient solution treatment. Microbial biofertilizers are integral components of salinity-resilient hydroponic systems, as they target key physiological functions associated with ion imbalance, osmotic stress, and oxidative stress. The operational suitability of recirculating systems depends on their ability to withstand hydraulic turbulence, carbon availability, and periodic sanitation events.

*Bacillus* species are effective because of their stress resistance, ability to re-establish after washout, and capacity to colonize inert matrices and root surfaces. Bacterial–fungal consortia outperform single-strain inoculants under variable EC conditions through nutrient solubilization, phytohormone regulation, and enhanced rhizosphere stability. The results in Table 3, including improvements in yield, biomass, ionic regulation, antioxidant function, and metabolic stability, indicate that microbe-based technologies are viable for maintaining hydroponic crop performance under saline conditions.

### 4.2. Limitations and Operational Risks

The application of microbial biofertilizers in recirculating hydroponic systems has serious limitations that affect their reproducibility, safety, and reliability. Studies have shown that in inert substrate systems, root-attached populations of beneficial bacteria, such as Bacillus spp. and Pseudomonas, decline by 2 log_10_ CFU per mL within 24 h under continuous flow conditions [92]. This washout effect negates the benefits of colonization when the circulation of the nutrient solution is high.

Biofilm formation poses operational hazards. Multispecies biofilms develop on pipe walls, NFT channels, emitters, and reservoirs, increasing hydraulic resistance and reducing the dissolved-oxygen supply [93]. Commercial systems showed microbial surface loads of 7.3–7.5 log CFU/cm^2^ on PVC surfaces within weeks, which correlated with channel clogging [94]. Sanitation measures for pathogen control conflict with inoculant viability issues. Routine sanitization protocols are required to suppress these pathogens. Sodium hypochlorite at 50 ppm reduced biofilm loads to <1 log CFU/cm^2^ within 12 h, whereas on the commercial scale, the required NaOCl dose was increased to 500 ppm [94]. Such doses cause phytotoxicity and disrupt inoculant viability.

Pathogen proliferation is critical in recirculating systems. Biofilm-forming non-pathogenic taxa enhance the attachment of human pathogens, such as *Salmonella*, to PVC surfaces [94]. Pathogens can spread throughout a system through water circulation [93]. The lack of ecological buffering, compared to soil systems, limits stability. Hydroponic root zones lack bioorganic colloids and native microbiota that would buffer inoculant fluctuations [92]. Thus, the performance of inoculants is often short-lived and system-specific.

Although microbial biofertilizers have shown potential in controlled experiments, their application in hydroponics remains limited by colonization instability, biofilm risks, sanitation-inoculant incompatibility, and vulnerability to pathogens. These limitations must be addressed through improved formulation, optimized sanitation, and risk management before commercial adoption. Although quantitative outcomes have shown plant-level benefits, inconsistent reproducibility across facilities highlights the need to understand microbe-plant-system interactions. Inoculant success depends on compatibility with system hydraulics, root exudation, substrate physicochemistry, and sanitation. We integrated these engineering and ecological dimensions to understand why certain inoculants thrive while others fail through washout, oxidation, or biofilm instability.

## 5. Microbe–Plant–System Interactions in Hydroponics Systems

### 5.1. Formulation and Encapsulation Engineering

#### 5.1.1. Delivery Modes and Inoculation Strategies

In hydroponics, inoculant performance is determined by system configuration and hydraulic flow, which regulate the residence time, mass transfer, and root–microbe interactions. Three delivery approaches are typically used: seed biopriming, pre-transplant root dipping, and direct inoculation into nutrient reservoirs, which vary in colonization kinetics, durability, and function. In floating and NFT systems, inoculation with *B. subtilis* at 10^4^–10^5^ CFU mL^−1^ increased lettuce shoot mass by 15–25%, enhanced net photosynthesis by 95%, and improved nutrient accumulation, indicating that moderate bulk-phase titers achieve stable rhizoplane colonization in recirculating systems [30]. Similarly, in floating hydroponic lettuce under 20 mM NaCl, commercial *Bacillus*-based biostimulants improved growth and salinity tolerance compared to untreated plants [39]. In addition, studies have shown that the efficiency of microbial inoculation depends on hydraulic dynamics, with high-flow systems reducing microbial retention. Single-point dosing often results in a rapid decrease in microbial abundance, necessitating controlled application to maintain stable microbial populations.

Circulation and oxidative exposure reduce microbial survival, formulation stability, and durability; thus, repeated dosing is required for a sustained activity. Studies have shown that periodic re-inoculation maintains microbial performance more effectively than a single-dose application. Encapsulation using alginate or carrier matrices improves viability by creating protected microsites that reduce oxidative stress and hydraulic forces [43,44]. In NFT and DWC systems, *Bacillus* strains have shown better performance than fungal inoculants for NUE and plant productivity [95]. Therefore, the integration of reservoir-based inoculation with scheduled re-dosing and protective encapsulation matrices has proven effective for improving biomass production, photosynthetic efficiency, and nutrient acquisition, which promotes stable microbiomes under recirculating hydroponic conditions.

#### 5.1.2. Compatibility with Nutrient Solution and Disinfection Practices

Disinfection and nutrient management are central to hydroponic production but can reduce the survival and activity of beneficial inoculants. UV irradiation, ozone exposure, and hydrogen peroxide-based sanitation inhibited phytopathogens but damaged the inoculated microbial populations. UVA-LED exposure at 40–60 mJ cm^−2^ causes a 3–5 log reduction in microbes in nutrient solutions [40,96]. Electrochemically generated H_2_O_2_ at 200 mg L^−1^ reduced bacterial and fungal counts by 93% and 81%, respectively, within 60 min, with extended exposure achieving >97% reduction [41]. In baby chard hydroponics, ozone treatment at 0.5–2.0 mg L^−1^ inhibited pathogens but induced oxidative stress in the roots and leaves. Ozone microbubble systems eliminate bacteria within 5 min while inducing viable-but-non-culturable states [42,97]. Therefore, inoculation timing should follow oxidant dissipation or be confined to root zones isolated from sanitation loops to facilitate colonization without causing oxidative damage. In addition to disinfection, nutrient chemistry also affects compatibility; solutions with a pH above 6.5 precipitate phosphate and Fe-chelates, reducing nutrient availability and microbial performance [98]. Certain PGPR produce siderophores, increasing ferric ion solubility by 25–35% under alkaline conditions and promoting chlorophyll synthesis and Fe nutrition [99].

Encapsulation improves microbial persistence in hydroponic solution. For instance, encapsulated *B. amyloliquefaciens* showed 80% viability after 48 h of exposure to 1 mg L^−1^ H_2_O_2_, whereas free cells exhibited <5% survival, with viable propagules released for 10–14 d in recirculating systems [43,44]. These microformulations limit oxidant penetration, reduce ionic strength, and promote stable colonization and microbial activity in recirculating solutions. Therefore, protective encapsulation and compatible inoculation timing are essential for maintaining functional microbial biofertilizers in hydroponic systems.

### 5.2. System-Level Operational Constraints

#### 5.2.1. Colonization Dynamics and Root–Microbe Adhesion

In soilless cultivation, plant–microbe interactions differ from those in soil-based systems, as the rhizosphere lacks organic colloids and is dominated by inert surfaces, which modify microbial colonization and nutrient exchange. The performance of biofertilizers depends on their ability to colonize inert substrates and maintain stable rhizoplane populations. Key beneficial strains, including *B. subtilis*, *P. putida*, and *Azospirillum brasilense*, utilize chemotactic responses to root-derived sugars, amino acids, and organic acids to establish colonization of the root [100]. In addition, the physicochemical complexity of soilless root zones affects the distribution and durability of microbial inoculants, which requires stronger adherence mechanisms. In hydroponics, root exudates diffuse into circulating nutrient solutions, and dilution limits microbial accumulation at the root–solution interface of the plant. Total carbon exudation in soilless cultivation can exceed that in soil-based systems by more than 25% of root carbon allocation [101].

Sustained colonization requires adhesive traits, such as EPS secretion and biofilm formation. *B. subtilis* lacking *eps* and *tasA* had 60% lower root surface occupancy within 7 days than wild-type strains [102]. These results show that adhesion-associated proteins and polysaccharides determine microbial durability on inert substrates, where mechanical shear and continuous nutrient flow limit colonization efficiency.

Under recirculation, single-strain *B. subtilis* inoculation decreased by 2 log_10_ CFU per seedling within 24 h, whereas co-inoculation recovered the population by 1–1.5 log_10_ CFU [103]. In nutrient-film lettuce, *B. subtilis* inoculation at 7.8 × 10^3^–3.1 × 10^4^ CFU mL^−1^ increased shoot biomass by 22–25% and enhanced photosynthetic rates by 95% [30]. The performance of inoculants depends on chemotactic recruitment, EPS-mediated biofilm formation, and strain-specific adhesion traits. Future biofertilizer formulations should prioritize high-*eps*- and *tasA*-producing strains and incorporate colonization screening protocols for hydroponic systems.

#### 5.2.2. Root Exudation and Nutrient-Mediated Microbial Selection

In hydroponics, root exudation is regulated by the plant genotype and nutrient formulation, generating biochemical gradients at the root–solution interface. Circulating solutions accelerate diffusion and dilution, reducing exudate residence time compared to soil systems. Approximately 15–35% of fixed carbon may be released as root metabolites, depending on the species, growth stage, and medium composition [101,104]. Under saline conditions, roots increase the efflux of compatible solutes and carbon compounds, including proline, sugars, amino acids, phenolic acids, and organic acids, to reduce stress. Studies have shown a 1.8- to 3.2-fold increase in carbon exudation under salt stress [105]. These metabolites maintain osmotic balance and act as chemoattractants for rhizobacteria to colonize the root. In addition, the recirculation of nutrient solutions in hydroponics intensifies the variability of root-derived metabolites, affecting the ability of microorganisms to detect and respond to signaling molecules.

Microbial utilization of exudates is taxonomically selective. For example, *Pseudomonas fluorescens* exhibits chemotaxis towards malate, citrate, and succinate, developing up to 1.6-fold higher biofilm density on these substrates [106]. Similarly, *B. subtilis* and *Bacillus amyloliquefaciens* metabolize amino acids and osmoprotectants, including glutamate, choline, and glycine betaine, maintaining a population of 10^6^–10-CFU mL^−1^ in hydroponic systems [30,107]. This utilization capacity, influenced by MarR-type transcriptional repressors, supports balanced uptake, biosynthesis, and osmoregulatory stability under high-ionic conditions [107].

Metagenomic studies have shown that organic and amino acids influence microbial community formation and promote resilience to abiotic stress [108]. This integration between plant exudates and microbial assimilation stabilizes hydroponic microbiomes, where chemotaxis, substrate specificity, and nutrient-dependent colonization sustain beneficial populations under continuous flow, improving crop performance in soilless environments.

#### 5.2.3. Substrate and System-Specific Microbial Interaction

Hydroponic systems differ in nutrient hydraulics and substrate physicochemical properties, which regulate microbial attachment, moisture retention, and oxygen availability at the root–substrate interface of the plant. Compared with inert rockwool, coconut coir has a higher cation-exchange capacity and distinct hydraulic traits, retaining more water but exhibiting a lower saturated hydraulic conductivity. Perlite is inert, highly porous, and exhibits very high hydraulic conductivity, providing rapid drainage and aeration [109]. These characteristics create substrate-specific microbial niches that influence the colonization, durability, and functional activity of the microbiota. In addition, the contrasting sorption behavior, pore geometry, and aeration profiles of these substrates exert regulatory effects on the microbial population structure, as microsites for adhesion and moisture films determine microbial durability under recirculating conditions. Mineral substrates, such as rockwool, depend on solution-borne propagules to establish microbiomes, whereas organic substrates, such as coconut coir, contain organic matter and reactive surface chemistry that promotes diverse and abundant microbial populations [110,111]. Studies have shown that coconut coir cultivation results in higher tomato biomass, nutrient uptake, and photosynthetic activity than rockwool, indicating that substrate–rhizosphere interactions extend beyond the physical water–air balance [112].

In hydroponic leafy vegetables, *Trichoderma harzianum* and *A. brasilense* improved lettuce nutrient content and yield, whereas *Azospirillum* alone increased the chlorophyll content and biomass [88,89]. These results suggest that organic matrices provide more adhesion sites than rapidly draining inert media because of their higher water-holding capacity and surface heterogeneity [109,110]. In NFT systems, excessive microbial growth increases hydraulic resistance and reduces dissolved oxygen availability, making flow rate management and oxygen supply critical when applying microbial inoculants to thin-film systems [6,113]. These substrate architectures regulate microbial durability and activity in these systems. Inert matrices provide hydraulic stability but have limited colonization capacity, whereas organic substrates enhance diverse microbial communities but require careful management to avoid anoxic conditions.

#### 5.2.4. Interactions with Plant Physiology

Microbial colonization in hydroponics influences plant phenotypes by modulating root architecture, hydraulic conductance, and rhizochemical conditions of plants. In NFT-grown lettuce, *B. subtilis* inoculation at 7.8 × 10^3^–3.12 × 10^4^ CFU mL^−1^ increased photosynthesis by 95%, water-use efficiency by 67%, and significantly increased shoot and root biomass and nutrient accumulation, including N, P, K, Ca, Mg, and S [30]. Microbial inoculation influences plant physiological processes, particularly under saline conditions, where water relations, pigment stability, and ion transport dynamics are limited. Under saline conditions (50 mM NaCl), PGPR inoculation increased the stomatal conductance by 58–189%, relative water content by 9–108%, and chlorophyll content by 4–10% [8]. Lettuce inoculated with *A. brasilense* and *T. harzianum* showed increases of 47% and 20% in root proliferation, respectively, with enhanced leaf biomass under optimal EC ranges (1.2–1.4 dS m^−1^) [88].

Fungal biostimulants, such as *Trichoderma* spp., enhance nutrient dynamics through the secretion of organic acids, improving phosphate solubilization and mobilization. AMF-inoculated plants showed 141% higher P-uptake efficiency than control plants [114]. Under saline hydroponics, *Trichoderma* and *Bacillus* strains modulate phytohormonal homeostasis involving the IAA, ABA, salicylic acid (SA), and jasmonic acid (JA) pathways, improving root development and osmotic balance [115]. These structural, physiological, and biochemical modifications enhance nutrient acquisition and biomass accumulation in inoculated plants under moderate salinity stress. Studies have shown that PGPR and *Trichoderma* consortia enhance photosynthesis, WUE, root proliferation, and nutrient uptake through P-mobilization and phytohormonal regulation. These results facilitate the integration of bacterial and fungal inoculants into saline-resilient hydroponic systems to stabilize plant performance under saline conditions.

#### 5.2.5. Operational Risks and Control Strategies

Although microbial biofertilizers improve plant performance, their use in recirculating hydroponics requires precise engineering and supervision. Biofouling and emitter clogging affect hydraulic stability, and biofilm development is correlated with reduced discharge in drip systems. Optimized water–fertilizer–gas management reduces clogging and increases the emitter lifespan by approximately 29% [116]. Microbial inoculants in closed-loop systems influence hydraulic behavior because biofilm formation on components can increase flow resistance and affect pressure distribution and nutrient delivery. UV-C disinfection reduces the planktonic microbial load in the circulating solution. In a 5 W UV-C reactor (400 L h^−1^), lettuce harvested 8–20 days earlier than controls, with chlorophyll-a increasing to 0.61 ± 0.03 mg g^−1^ compared to 0.40 ± 0.07 mg g^−1^ without UV-C [117]. Low-dose oxidants are suitable for sanitation when they are managed precisely. In greenhouse trials, chlorine dioxide below 0.7 mg L^−1^ prevented biofilm formation, whereas ultrasound or antibacterial tubing produced negligible benefits [118].

The durability of the residual microbiota in nutrient solutions is temperature-dependent. In modified Hoagland solution, *Salmonella javiana* persisted for 21 d with 4.0–4.7 log_10_ reductions at 30–37 °C, whereas *Listeria* species decreased more rapidly [119]. NFT systems have identified biofilm-forming strains that facilitate the attachment of *Salmonella* to PVC components [94]. Precision dosing systems can prevent overcolonization. Closed-loop fertigation models achieved R^2^ = 0.7–0.9 and 85–88% water savings, providing event-triggered inoculation [120]. AI-supported systems integrate sensor data to automate dosing cycles, suspend inoculation during oxidant spikes, and resume below compatibility thresholds [121,122]. These insights indicate that microbe-based strategies require structured control, sanitation protocols, and reliable sensor-mediated systems.

## 6. Formulations, Bioprocessing, Regulatory, and Market Regulations

The regulatory and market frameworks for microbial products are relevant when interpreted based on the operational vulnerabilities of hydroponic systems. As shown in Section 4.2 and Section 5.2, recirculating nutrient environments are prone to microbial washout, hydraulic obstruction, pathogen proliferation, and disinfection–inoculant incompatibilities, creating a regulatory profile that is distinct from that of soil agriculture. These constraints require hydroponic-specific regulation, in which microbial purity, pathogen exclusion, propagule concentration, and survivability are evaluated under actual recirculating conditions. Regulatory frameworks must be aligned with physicochemical realities and biological fluctuations that define soilless environments.

### 6.1. Formulation Stability and Delivery in Hydroponics Systems

Hydroponic nutrient solutions operate at EC levels of approximately 1–3 dS m^−1^ and lack organic matrices that buffer soil microbes. Continuous nutrient circulation and sanitation reduce the microbial residence time. Hydroponic formulations must combine halotolerance with delivery systems to ensure storage stability, controlled release, and sustained microbial performance under fluctuating ionic pressure. The dilute and circulating nature of hydroponic systems reduces microbial retention, necessitating the development of formulations that resist dispersive loss. The success of microbial biofertilizers depends on their structural robustness, tolerance, and capacity for sustained propagule release [123].

Shelf life varies according to microbial taxa and matrix architecture. Spore-based products maintain high viability, with *B. amyloliquefaciens* (CPA-8) showing no viability loss after 12 months under varied conditions, and cold storage at 4 °C affecting only visual characteristics [124]. Similarly, *B. subtilis* Bs006 maintained >90% survival in liquid and 85% survival in solid formulations after 12 months at 20–40 °C [125]. Alginate-based microgels containing *Trichoderma* retained ≥70% viability for 24 months, with 1.5 ± 0.3 mm beads yielding 1.5 × 10^8^ ± 0.2 × 10^8^ conidia mL^−1^ by day 9 [126]. Bioreactor-in-granule architectures maintain conidial viability for more than 24 months [127].

Encapsulation technologies mitigate hydroponic dilution by slowing propagule release and improving rhizoplane durability. Alginate matrices (2–3 mm) extend root residence time and reduce dispersive bursts. Encapsulated *Bacillus*–*Pantoea* inocula sustained root colonization for a longer duration than free cell suspensions [128]. Encapsulation, granulation, and spore-centric design are key strategies for maintaining microbial persistence under high-flow hydroponic conditions. In contrast, liquid formulations require frequent reapplication.

### 6.2. Carrier Matrix and Physicochemical Compatibility

The carrier matrix mediates the biological stability and physicochemical suitability of the microbial inoculants in hydroponic systems. Conventional soil carriers, including peat, vermiculite, talc, and biochar, provide protective microhabitats but generate particulate residues unsuitable for recirculating hydroponics because of emitter and filter clogging. Studies have shown a 25–35% reduction in emitter discharge within 21 d of particulate contamination [129]. Hydroponic formulations prioritize liquid or colloidal carriers that remain suspended without solid release. The particle-sensitive hydraulics of recirculating systems require carriers that remain chemically inert, structurally stable, and non-sedimenting because particulates impair the emitter function and nutrient distribution [129]. Liquid carriers, such as sterile phosphate buffers, molasses–glycerol mixtures, and oil-in-water emulsions, maintain osmotic potentials of −0.6 to −0.8 MPa to prevent plasmolysis during storage. Emulsified formulations have shown higher viability than aqueous systems, whereas oil- or lecithin-stabilized carriers improve shelf stability at room temperature [130,131]. These carriers provide osmotic buffering, minimize metabolic desiccation, and enhance membrane integrity during extended storage periods.

Encapsulation and nanocomposite carriers exhibit superior stability in hydroponics, where oxidative radicals, ionic fluctuations, and shear forces threaten microbial survival. *B. subtilis* encapsulated in alginate–bentonite beads with TiO_2_ nanoparticles maintained 4 × 10^9^ CFU g^−1^ at day 45 and achieved 90% disease inhibition compared to 60% using free-cell inocula [132]. Similarly, *Bacillus velezensis* in alginate–gelatin nanocomposite capsules released propagules for over 50 d, with 2.5-fold higher root colonization than unencapsulated cells [133]. Encapsulation efficiencies exceeding 90% and long-term viability preservation consistently demonstrated the capacity of alginate-, chitosan-, and nanoparticle-enriched matrices to resist oxidative damage, ionic stress, and hydraulic shear.

Polymeric and nanostructured carriers protect microorganisms from oxidative radicals, enhancing their residence time near the plant rhizoplane. The encapsulated formulations persisted for 20 days in hydroponic solutions, whereas non-encapsulated cells lasted for less than 5 days [132]. Protection against oxidative species and ionic fluctuations is crucial in the design of hydroponic fertilizers. Solutions with phosphate concentrations above 15 mM or Fe–EDTA concentrations above 20 µM destabilize colloidal systems, whereas chloride-rich solutions above 50 mM reduce the bacterial half-life by 50% [134]. *T. harzianum* tolerates EC values up to 8 dS m^−1^, whereas *P. fluorescens* viability decreases above 6 dS m^−1^, without osmoprotectants such as 1–2% trehalose or 0.5% PEG-4000 [135,136]. Carrier engineering has become crucial for inoculant performance, with nanocomposite- and osmoprotectant-enriched matrices enabling high encapsulation efficiency, 6–12 months of storage stability, and 20 days of near-root durability. These carriers form the basis for the development of next-generation hydroponic biofertilizers suitable for high-EC, low-carbon, and oxidation-prone environments.

### 6.3. Bioreactor Scale-Up and Process Optimization

Industrial-scale biofertilizer manufacturing for hydroponics requires efficient upstream and downstream bioprocessing. For Gram-positive inoculants, including *Bacillus* spp., stirred-tank aerobic fermentation remains the standard, with dissolved oxygen maintained above 30–50% to sustain aerobic metabolism. Bench- and pilot-scale fermentations on glucose–yeast extract or mineral-salt media yield 15–20 g DCW L^−1^, whereas high-cell-density strategies using two-stage feeding, oxygen enrichment, and foam control achieve titers exceeding 40 g L^−1^ [137,138,139]. Industrial fermentation systems have been optimized to sustain biomass productivity under controlled aeration–agitation regimes because oxygen transfer, shear sensitivity, and nutrient depletion are key scale-up bottlenecks. Medium optimization with regulated aeration at pH 7 generated spore titers near 1.3 × 10^9^ CFU mL^−1^ and sporulation efficiencies of 85–95%. Glucose, Mg^2+^, and yeast extract concentrations are key metabolic determinants of sporulation yield, enzyme activity, and metabolic flux [138,140,141].

For filamentous species, including *Trichoderma*, *Beauveria*, and *Metarhizium*, packed-column, rotating-bed, and air-lift reactors produce approximately 10^8^–10^10^ conidia g^−1^ within 4–5 d [142,143]. These systems allow for improved oxygen transfer, consistent nutrient distribution, and reduced shear stress, which are critical for maintaining structural integrity and sporulation efficiency. Continuous cultivation with foam fractionation reduces downstream operations, such as centrifugation and solvent treatment, with techno-economic assessments showing a 25% reduction in downstream costs, comprising 25–60% of the total production cost [144,145,146]. These integrated approaches reduce energy requirements, increase product recovery efficiencies, and improve operational feasibility.

Genetic engineering can improve process resilience and product performance. *TreS* overexpression enhances solute biosynthesis, *katE* increases oxidative stress tolerance, and *groESL* upregulation improves desiccation resistance, drying productivity, and long-term viability [147,148,149,150]. These enhancements stabilize microbial performance under high EC conditions, drying stress, and storage intervals. Therefore, bioprocess optimization, including reactor configuration, medium engineering, integrated processing, and genetic enhancement, is essential for developing scalable, cost-effective, and physiologically efficient microbial inoculants for hydroponic systems.

### 6.4. Shelf-Life and Storage Kinetics

The shelf life of microbial biofertilizers follows first-order decrease kinetics in cultivable units, expressed as Nₜ = N_0_e^–^ᵏᵗ, enabling the quantitative estimation of the destruction rate constant (k) from plate-count data using Arrhenius models [151]. Loss rates are formulation- and strain-specific, with *P. fluorescens* decreasing by 0.05–0.20 log_10_ CFU month^−1^ at 20–30 °C, depending on the carrier type, water activity, and stabilizer [130]. The storage behavior of hydroponic biofertilizers is influenced by inoculant traits and carrier system stability, with moisture, oxygen, and temperature affecting the degradation kinetics. Quantitative decay modeling is essential for predicting potency maintenance, particularly in fertigation systems.

Spore-forming *Bacillus* species show higher storage stability, with a decrease limited to <0.1–0.3 log_10_ CFU over 12 months at 25 °C, with several preparations showing no reduction over 6–12 months [152,153]. Refrigerated storage (4–8 °C) increases the storage duration 2- to 3-fold by inhibiting metabolic turnover and moisture-associated degradation pathways [151]. This thermal resilience reflects the capacity of spores to resist oxidative, hydrolytic, and osmotic destabilization, providing an advantage for hydroponic applications requiring intermittent dosing.

Freeze-dried powders packaged under vacuum with desiccants and maintained below 0.2–0.3% residual moisture retain ≥10^8^ CFU g^−1^ for 12–18 months and require overage, cryoprotectants/lyoprotectants, and controlled low-humidity packaging [152,153,154]. The incorporation of trehalose, skimmed milk proteins, and polyol protectants enhanced membrane stability during lyophilization, whereas oxygen-barrier laminates extended viability during storage.

Regulatory systems require potency verification, and EU regulations include microbiological stability documentation [155,156]. However, post-dilution viability in high-EC nutrient solutions and survival in recirculating reservoirs remain unassessed, despite their relevance to hydroponic cultivation. The environmental performance of inoculants under soilless conditions remains largely uncharacterized, highlighting the need for system-specific protocols [25,39].

### 6.5. Regulatory Systems and Quality Standards

The global regulatory environment for microbial biofertilizers is shifting toward harmonized systems that integrate safety, quality, and compliance. Within the European Union, Regulation (EU) 2019/1009 classifies microbial fertilizing materials under Component Material Category (CMC) 7 and requires conformity with the PFC and CMC specifications for CE certification. Industrial practices apply benchmarks of ≥10^8^ CFU g^−1^, with strict contaminant limits, including the absence of fecal coliforms, enteric pathogens, and *Salmonella*, supported by stability datasets [157]. The European framework emphasizes traceability, reproducibility, and validated microbial identity, reflecting a movement toward quantifiable quality assurance. Microbial biofertilizers must comply with contamination thresholds and provide evidence of their viability, stability, and manufacturing consistency. These requirements are critical in hydroponic systems, where washout, oxidant exposure, and biofilm-associated risks intensify the consequences of inadequate quality.

In India, the Fertilizer Control Order (FCO) 1985, updated through the Fifth Amendment Order 2021, regulates microbial products, including “biofertilizers” and “biostimulants.” The system specifies microbial categories, such as *Rhizobium*, *Azospirillum*, and *Trichoderma*, using CFU thresholds, moisture and pH criteria, and analytical methods for certification [158]. However, neither the EU nor Indian regulations incorporate hydroponic-specific requirements, such as post-dilution viability, high-EC tolerance, or compatibility with UV and peroxide sanitation. Product approval relies on ISO/IEC 17025 validation, rather than system-level performance [159]. This gap is significant because hydroponic environments expose inoculants to conditions that alter their survival and function.

The draft FAO “Biofertiliser Quality Protocol” proposes including hydroponic metrics, such as EC tolerance, soilless media, and oxidative disinfection compatibility, indicating alignment with system-specific standards. These evolving standards reflect a shift toward evaluation systems in which microbial resilience and compatibility under hydroponic conditions are mandatory. Market entry requires operationally relevant validation criteria beyond soil-based assessments, including bioprocess engineering, performance testing, and quality standards. These criteria address mechanistic limitations, ensuring that inoculants can withstand ionic stress, oxidative loads, and low-carbon conditions in recirculating systems.

The regulatory landscape functions as a structural extension of mechanistic and operational constraints. By linking inoculant approval criteria to hydroponic-specific risks, such as pathogen co-colonization, VBNC states after sanitation, strain instability, and substrate-dependent adhesion, regulations ensure that commercial biofertilizers meet the demands of recirculating systems. This alignment enables the transition from experimental applications to scale-ready hydroponic biostimulant technologies, integrating mechanistic understanding with regulatory design.

## 7. Future Perspectives

The next frontier for hydroponic biofertilizers is the integration of digital agriculture and AI-mediated monitoring systems. In modern hydroponics, nutrient management uses automated dosing systems that regulate pH, EC, nutrient solution temperature, and volume in real time. These systems are divided into feedback control and predictive analytics, with pH and EC as the key monitored variables [Sulaima]. Incorporating microbial biofertilizers into such systems would allow for the control of inoculation, nutrient levels, and salinity mitigation within the same optimization loop.

IoT-based sensors and AI-mediated systems provide a framework for transforming microbial inoculation from static to dynamic data-responsive fertigation-management systems. The iPONICS platform demonstrated a cloud-connected greenhouse in which sensors were linked to an IoT gateway and fuzzy logic rules for irrigation [160]. Future fertigation systems may incorporate microbial performance indicators, providing AI-based engines to optimize inoculation based on EC, pH, dissolved oxygen, and crop conditions. This AI-aligned dosing compensates for physicochemical and hydraulic limitations, preventing oxidant spikes and shear events.

Digital twins represent an approach for integrating microbial processes into controlled environment agriculture. These systems link sensor networks with virtual representations and use reinforcement learning to optimize the monitoring process [161]. Extending digital twins to incorporate microbe-plant-ion interactions would enable the testing of inoculation strategies before their application [162]. Such “microbe-aware” digital twins are suitable for hydroponic salinity scenarios, where EC, osmotic potential, and microbial activity must be interpreted as integrated phenomena.

Smart formulation technology provides digital integration of biofertilizers. Nano-biofertilizer formulations using biopolymers, such as alginate, chitosan, and cellulose, can be engineered into hydrogels and microcapsules for the controlled release of nutrients and microbial cells [163,164]. Encapsulated microbial inoculants improve stress tolerance and are suitable for smart farming [163]. These matrices overcome ionic stress and oxidative loading in recirculating nutrient systems.

Polymeric agrochemicals provide the precise release of active ingredients while reducing the input frequency and environmental loading [164]. These advances have led to the development of smart bioinoculant systems with programmable release kinetics and digital fertigation compatibility. Alginate-based matrices have successfully immobilized *Bradyrhizobium* and PGPB, maintaining their viability during storage and enabling controlled release [165]. Alginate microbeads with flavonoids enhance symbiosis in *Arachis hypogaea*, demonstrating climate-resilient properties [165]. Chitosan-coated microcapsules exhibited pH-responsive behavior suitable for halotolerant biofertilizer consortia in hydroponic solutions [166].

These developments have enabled the use of instrumented biofertilizer microcapsules with integrated colorimetric or electrochemical reporters to indicate viability and metabolic status. Although not widely implemented, underlying materials science and release models have been established [164,166]. Combined with IoT sensing, AI controllers, and digital twins, these smart biofertilizer systems can transform hydroponic inoculation from batch-wise application to continuous, data-guided biomanagement aligned with the Industry 5.0 principles of human-centric, resilient, and sustainable CEA.

## 8. Conclusions

Salinity remains a persistent constraint in hydroponic production, as continuous recirculation and limited drainage enhance ionic accumulation and destabilize root-zone physiology. Although microbial biofertilizers show promise in improving plant performance under saline conditions, several limitations limit their commercial application. The stability of inoculated microbial populations, their compatibility with fluctuating EC environments, and their persistence under sanitation regimes remain poorly understood. Most studies depend on short-term trials and single-strain inoculations, providing limited insight into the long-term dynamics of NFT, DWC, and substrate-based hydroponics.

Key knowledge gaps include quantifying inoculant survival under continuous flow, defining biofilm safety thresholds, assessing pathogen interactions, and developing models that integrate plant physiology and nutrient solution chemistry. Priority research areas include multi-strain consortium design, hydroponic-specific formulations, and monitoring tools for microbial viability and root colonization in plants. Significant barriers persist: formulations lack standardized shelf-life benchmarks, oxidizing disinfectants are often incompatible with inoculants, and no regulatory guidelines exist for microbial products in recirculating hydroponic systems.

For growers, practical implementation requires reliable inoculant delivery systems, compatibility with automated schedules, and evidence of stable yields across production cycles. Progress requires the integration of microbial biotechnology with system engineering, digital monitoring, and risk management. Addressing these gaps is essential for translating promising laboratory results into commercially scalable strategies for the hydroponic production of salinity-resilient crops.

## Figures and Tables

**Table 1:** Effects of salinity intensity on morpho-physiological growth parameters, nutrient assimilation responses, and yield performance of crops cultivated in hydroponic systems.

Crop Species	Salinity Threshold in Hydroponics Nutrient Solution (EC/NaCl)	Plant Growth Effects	References
Tomato (*Solanum lycopersicum* L., cherry cv. ‘Pepe’)	EC: 0.78, 0.91, 1.10, 1.26, 1.41, and 1.58 dS m^−1^	Biomass and chlorophyll were reduced only at 1.58 dS m^−1^. The yield decreased at ≥1.41 dS m^−1^. Increased Brix and titratable acidity with increasing EC; evapotranspiration decreased at highest EC; WUE unchanged (0.78–1.58 dS m^−1^)	[46]
Lettuce (*Lactuca sativa* L.)	10, 20, 40, and 60 mM L^−1^ NaCl	Shoot biomass decreased by >2-fold at 60 mM NaCl, and dry matter increased by 32%. Decreased leaf N (11%), K (35.7%), and Mg (24.5%); increased Na 24-fold; Na^+^:K^+^ ratio shifted from 76:1 to 2.6:1; RWC 6.2%. PSII parameters decreased, and moderate–high NaCl reduced yield, dry matter, and leaf nitrate.	[47]
Spinach (*Spinacia oleracea* L.)	EC: 0, 4, 6, 8, 10, and 12 dS m^−1^	Root length decreased from 14.2 cm to 5.9 cm at 12 dS m^−1^; shoot length decreased at 8 dS m^−1^. Leaf area decreased from 39.5 cm^2^ to 9 cm^2^ at 12 dS m^−1^. RWC was stable until 6–8 dS m^−1^, then dropped to 32% at 12 dS m^−1^. Increased proline, SOD, and CAT levels at higher EC.	[48]
Cucumber (*Cucumis sativus* L.)	50–100 mM NaCl	NaCl (50–100 mM) reduced leaf area, root growth, chlorophyll, and PSII efficiency (Fᵥ/Fₘ). Decreased CO_2_ assimilation, N, K, and Mg, and increased Na and Cl^−^. 13% yield loss per dS m^−1^ above 2.5 dS m^−1^.	[49]
Bell pepper/sweet pepper (*Capsicum annuum* L., cv. ‘Machai’)	EC: 4.0, 4.5, 5.0, and 5.5 dS m^−1^	Rockwool substrate: Plant height, leaf area, shoot FW/DW; yield 14.7 kg to 8.7 kg slab^−1^ decreased at 5.5 dS m^−1^ (40% reduction). Coconut coir substrate: Moderate reduction but yield stable (11.1–16.2 kg slab^−1^, 4.0–5.5 dS m^−1^).	[50]
Strawberry (*Fragaria* × *ananassa* Duch., cv. ‘Primoris’)	2 mM and 7 mM NaCl	At 7 mM NaCl, the yield and firmness were maintained, SSC increased, and flavor improved. Increased polyphenol and antioxidant activities (ABTS, DPPH). Increased Flavan-3-ols, anthocyanins, and total phenolics under 7 mM NaCl.	[51]

**Table 2:** Microbial inoculants produce hormones and secondary metabolites that mitigate salinity stress and promote crop growth.

Microbial Inoculant	Phytohormone Production	Salinity Levels	System	Host Crops	Outcomes	Reference
*Pseudomonas* sp. UW4	Aminocyclopropane-1-carboxylic acid (ACC)-deaminase and trehalose	200 mM NaCl	Pot (peat moss substrate)	Tomato	Wild-type UW4 significantly increased shoot and root length, total dry weight and chlorophyll content vs. salt-stressed control; double mutant (acdS/treS) lost most protective effect, indicating combined role of ACC-deaminase and trehalose in salt tolerance and growth promotion.	[74]
*P. aeruginosa* GKP KS2_7 and *Bacillus subtilis* MBD133	ACC-deaminase, indoleacetic acid (IAA), exopolysaccharides (EPS), siderophores	500 mM	Pot (soil)	Pea	Seed bacterization with ACC-deaminase-positive strains reduced stress-induced ethylene, increased root and shoot length, fresh and dry biomass, chlorophyll, soluble sugars and antioxidant enzyme activities; proposed as ACC-deaminase-based biofertilizers for saline soils.	[75]
*Bacillus safensis* PM22	EPS, IAA, siderophores, ACC-deaminase	0, 180, 240, 300 mM NaCl	Pot (soil)	Maize	Inoculation improved root and shoot length, biomass, chlorophylls, carotenoids, relative water content, K^+^/Na^+^ homeostasis and antioxidant enzymes (superoxide dismutase (SOD), catalase (CAT), ascorbate peroxidase (APX), glutathione reductase (GR) and peroxidase (POD)), while decreasing H_2_O_2_, MDA, electrolyte leakage, glycine betaine and proline; strong enhancement of salt tolerance index.	[76]
*Rahnella aquatilis* JZ-GX1	Volatile organic compounds (VOCs)—2,3-butanediol	150 mM NaCl	In vitro (agar plates)	Black locust	VOCs from JZ-GX1 increased plant height, biomass, lateral root formation and photosynthetic performance; reduced Na^+^ accumulation and improved K^+^/Na^+^ ratio and antioxidant enzyme activities, clearly enhancing salt tolerance without direct root colonization.	[72]
*Bacillus amyloliquefaciens* FZB42	VOCs: 2,3-butanediol	100 mM NaCl	In vitro (agar plates)	*Arabidopsis thaliana*	FZB42 VOCs enhanced plant biomass, chlorophyll and root system architecture under NaCl; improved ion homeostasis and antioxidant status. Transcript profiling indicated modulation of salt-responsive genes, demonstrating VOC-mediated induction of salt tolerance.	[77]
*B. subtilis* GB03	VOCs, including 2,3-butanediol and 3-hydroxy-2-butanone (acetoin)	0.075 mM NaCl	Pot (soil)	*A. thaliana*	GB03 VOCs increased shoot and root biomass and reduced Na^+^ accumulation in tissues compared with NaCl-stressed controls; improved water status and ion homeostasis, establishing 2,3-butanediol as a central VOC signal in salinity and drought tolerance.	[78]
*Bacillus altitudinis* WR10	ACC-deaminase, IAA, phosphate-solubilising organic acids	12% NaCl	Hydroponics	Wheat	WR10 inoculation increased seed germination and seedling growth under salt stress; improved nutrient uptake and biochemical stress markers. ACC-deaminase and IAA production under saline conditions are directly associated with improved plant performance.	[79]
*Bacillus pumilus* FAB10	EPS, biofilm	75–500 mM NaCl	Pot (soil)	Wheat	FAB10 formed efficient biofilms under salinity, limited Na^+^ uptake and enhanced shoot and root growth, leaf area and biomass; higher K^+^/Na^+^ ratios and improved physiological traits showed that EPS-rich biofilms function as a salinity-mitigating biostimulant.	[80]
*P. aeruginosa* PF23 (fluorescent pseudomonad)	EPS, IAA, siderophores, hydrogen cyanide (HCN), lytic enzymes	2000 mM NaCl	Pot (soil)	Sunflower	EPS-producing PF23 improved sunflower growth and yield and reduced incidence of *Macrophomina phaseolina* under saline conditions; EPS-defective mutant lost much of the growth-promoting and stress-ameliorating effect, directly implicating EPS as a key metabolite in salinity mitigation.	[81]
*Pseudomonas*, *Burkholdera*, *Bacillus*, *and Arthrobacter*	IAA, siderophores, EPS	100 and 200 mM NaCl	Pot (coco peat, perlite, peat moss, zeolite mix)	Wheat	Selected halotolerant strains (e.g., isolates ALT29, ALT43) significantly increased shoot and root biomass, chlorophyll, relative water content and K^+^/Na^+^ ratio under salt stress. IAA and EPS production under salinity are specifically associated with improved performance.	[82]
*Trichoderma harzianum T34*	IAA, abscisic acid (ABA), and cytokinin (dihydrozeatin)	300 mM NaCl	Pot (coco peat)	Tomato	Seed coating with T34 increased growth in unfertilized plants and maintained gas-exchange under moderate salinity; affected expression of SOS1, ABA-, ethylene- and salicylic acid-pathway genes, indicating hormone-network reprogramming associated with salt adaptation PMC^+^1	[83]
*Trichoderma viride*	Phenolics, flavonoids, proline and antioxidant enzymes (CAT, APX, PPO, POD)	50 and 100 mM NaCl	In vitro (agar plates)	Tomato	Under 50–100 mM NaCl, T. viride increased total antioxidant capacity, raised proline by ~20%, enhanced CAT, APX and PPO activities (up to ~60–75%), and reduced MDA and H_2_O_2_ compared with salt-stressed controls, improving shoot/root growth and water status SpringerOpen	[84]
*Serendipita indica*	Signaling hormones (ABA, JA/SA), antioxidant enzymes (CAT, APX, PPO, POD)	150 and 300 mM NaCl	Substrate-based hydroponics (sand culture)	Tomato and barley	In tomato, *S. indica* inoculation under 50–150 mM NaCl increased plant height by ~43%, total dry weight by ~69% and chlorophyll by ~48%, while reducing MDA and ROS through increased antioxidant enzymes and an improved AsA–GSH cycle; In barley, *S. indica* increased abundance of photosynthetic proteins and shifted leaf proteome towards energy metabolism and detoxification under 300 mM NaCl	[85,86]
*Dunaliella salina*	EPS, uronic acids, phenolics and SOD, POD, CAT	50 and 100 mM NaCl	Pot (soil/peat mixture)	Tomato	EPS application increased shoot/root biomass, chlorophyll, phenolics and antioxidant enzyme activities; improved K^+^/Na^+^ ratios and reduced MDA and Na^+^ accumulation, significantly attenuating salinity-induced growth inhibition and membrane damage	[87]

**Table 3:** Quantitative effects of beneficial microbial inoculants on crop performance in hydroponic systems under salinity stress.

Microbial Inoculants	Host Crop	Salinity Level in Nutrient Solution	Outcomes	Reference
*Bacillus* spp.	Lettuce (floating hydroponic)	0 and 20 mM NaCl	Increased plant fresh weight (FW) (17.8–46%) and root FW (33.4%). PGPR maintained shoot FW and yield under 20 mM NaCl at levels comparable to the non-saline control, whereas uninoculated plants showed shoot FW reduction (17–23%) and yield loss (27%).	[39]
*Bacillus subtilis* (10^9^ mL^−1^), *B. megaterium* (10^9^ mL^−1^), *P. fluorescens* (10^10^ mL^−1^)	Lettuce (floating hydroponic culture)	50 mM NaCl	Increased yield (30–75%), FW (33–75%), leaf area (61–80%), leaf chlorophyll (4–10%), relative water content (9–107%), ion uptake (K^+^ 109%, Ca^2+^ 15–56%, Mg^2+^ 23–54%), and antioxidant enzymes (CAT, APX, GR up to 283%). Decreased Na^+^ accumulation (41–70%) and lipid peroxidation (MDA 26–42%).	[8]
*Glomus* sp.	Lettuce (DWC hydroponic)	0, 40, 80, 120 mM NaCl	Increased leaf area (59.3%) and shoot FW (72.5%) under 80 mM NaCl compared with uninoculated plants. AMF partially restored growth parameters under salinity, mitigating the salt-induced reduction in leaf area and FW at 80 mM NaCl.	[18]
*Trichoderma harzianum*, *A. brasilense*	Lettuce (NFT hydroponic)	EC levels: 1.2 and 1.4 dS m^−1^	Increased lettuce FW and leaf yield, with the highest yield improvement (28%) observed consistently in both EC levels Co-inoculation enhanced the uptake of K, P, Ca, Mg, Fe, Mn, Cu and Zn	[88]
*A. brasilense* strains AbV5 and AbV6	Lettuce (NFT hydroponic)	EC levels: 1.3–1.7 dS m^−1^	Increased leaf yield (15–25%), leaf number, chlorophyll content, and nutrient accumulation (shoot Ca: 109%, Mg: 74%, S: 69%), and enhanced N, P, K uptake	[89]
*B. subtilis* CCTB04 (7.8 × 10^3^–62.4 × 10^3^ CFU mL^−1^)	Lettuce (NFT hydroponic)	EC levels: 1.3–1.7 dS m^−1^	Increased leaf number (19–20%), shoot FW (22–25%), leaf yield (18–25%), net photosynthesis (95%), intercellular CO_2_ (30%), and water-use efficiency (67%).	[89]
*Chlorella vulgaris*	Lettuce (floating hydroponic system)	EC levels: 1.5–2 dS m^−1^	*C. vulgaris* enabled a 40–60% reduction in mineral fertilizer input with no yield loss, while increasing shoot FW, leaf area, N content, and chlorophyll concentration.	[90]
*Bacillus*, *Glomus*, *Lactobacillus*, *Pseudomonas*, and *Trichoderma* spp.	Pak choi (DWC hydroponic)	0, 40, 80, 120 mM NaCl	At 80 mM NaCl, the microbial consortium showed the greatest leaf area, and at 120 mM NaCl, it showed higher leaf chlorophyll and anthocyanin and less reduction in leaf area and FW than uninoculated controls.	[18]
*Bacillus* spp.	Cucumber seedlings (DWC)	75 mM NaCl	Increased root DW, leaf area, and root length and reduced shoot Cl^−^ accumulation	[91]

## Data Availability

No new data were created or analyzed in this study. Data sharing is not applicable to this article.

## Abbreviations

The following abbreviations are used in this manuscript:

EC	Electrical conductivity
NFT	Nutrient film technique
DWC	Deep water culture
PGPM	Plant growth-promoting microorganisms
PGPB	Plant growth-promoting bacteria
SOD	Superoxide dismutase
CAT	Catalase
APX	Ascorbate peroxidase
GR	Glutathione reductase
POD	Peroxidase
ACC	Aminocyclopropane-1-carboxylic acid
NUE	Nutrient-use efficiency
ROS	Reactive oxygen species
RWC	Relative water content
AMF	Arbuscular mycorrhizal fungi
MDA	Malondialdehyde
ABA	Abscisic acid
TFs	Transcription factors
SA	Salicylic acid
JA	Jasmonic acid
